# Novel viral and microbial species in a translocated Toutouwai (*Petroica longipes*) population from Aotearoa/New Zealand

**DOI:** 10.1186/s42522-022-00072-z

**Published:** 2022-10-12

**Authors:** Rebecca K. French, Zoë L. Stone, Kevin A. Parker, Edward C. Holmes

**Affiliations:** 1 Sydney Institute for Infectious Diseases, School of Medical Sciences, The University of Sydney, Sydney, NSW 2006 Australia; 2 Zoology and Ecology Group, School of Natural Sciences, Massey University, Palmerston North, New Zealand; 3 Parker Conservation Ltd, 549 Rocks Road, Nelson, New Zealand

**Keywords:** Translocation, Virus, Metatranscriptomics, Robin, Disease emergence

## Abstract

**Background:**

Translocation is a common tool in wildlife management and its implementation has resulted in many conservation successes. During translocations, any associated infectious agents are moved with their wildlife hosts. Accordingly, translocations can present a risk of infectious disease emergence, although they also provide an opportunity to restore natural infectious communities (‘infectome’) and mitigate the long-term risks of reduced natural resistance.

**Methods:**

We used metatranscriptomic sequencing to characterise the cloacal infectome of 41 toutouwai (North Island robin, *Petroica longipes*) that were translocated to establish a new population within the North Island of New Zealand. We also screened for pathogenic bacteria, fungi and parasites.

**Results:**

Although we did not detect any known avian diseases, which is a positive outcome for the translocated toutouwai population, we identified a number of novel viruses of interest, including a novel avian hepatovirus, as well as a divergent calici-like virus and four hepe-like viruses of which the host species is unknown. We also revealed a novel spirochete bacterium and a coccidian eukaryotic parasite.

**Conclusions:**

The presumably non-pathogenic viruses and microbial species identified here support the idea that most microorganisms likely do not cause disease in their hosts, and that translocations could serve to help restore and maintain native infectious communities. We advise greater surveillance of infectious communities of both native and non-native wildlife before and after translocations to better understand the impact, positive or negative, that such movements may have on both host and infectome ecology.

**Supplementary Information:**

The online version contains supplementary material available at 10.1186/s42522-022-00072-z.

## Background

Conservation translocations (hereafter ‘translocations’), in which organisms are moved from one location to another [1] for the purposes of conservation, are a common tool in wildlife management. Successful translocations can create a new population or augment an existing one, with the overall aim of reducing the risk of species extinction [2, 3]. Since the first globally documented translocation of kākāpō (*Strigops habroptilus)* in 1895 in New Zealand [4, 5], translocations have played a pivotal role in restoring biodiversity and preventing species extinctions. This technique has been responsible for many conservation successes around the world, including bringing the Arabian oryx (*Oryx leucoryx*) and Californian condor (*Gymnogyps californianus*) back from the brink of extinction [6, 7].

When organisms are moved, they will also transport any associated viruses, bacteria, fungi and parasites within them [8]. Translocations therefore alter the community ‘infectome’ (i.e., all infectious agents within an organism), including their ‘virome’ both at the source and destination locations [9]. This could be beneficial, by maintaining and restoring the natural infectome [10], or it may present a risk of disease emergence. The loss or decline of many host species has likely reduced the natural suite of parasites and other co-dependent species within these ecosystems. In some cases, intensive conservation measures including translocations have caused the extinction of their associated microbes [11], such that therefore restoring microbes to their previous geographic range could be beneficial for ecological conservation. It has been estimated, for example, that 2–4 species of lice have gone extinct as a result of conservation actions to save the host [11]. However, translocations could also increase the risk of disease emergence in the form of newly evolved pathogens or those that have increased their geographic spread, their host range or their pathogenicity [12]. Disease emergence could occur via transmission to or from the individuals being translocated in the new location, either to the same species (thereby increasing the pathogen’s range), or via cross-species transmission [13–15]. Animals may also experience considerable physiological stress during and after translocations [16], which may reduce their immune response, exacerbating current infections and increasing the likelihood of opportunistic infections [17]. The risk of disease emergence in translocations is increasingly being recognized [9, 14, 15, 18], as is the importance of viewing microbes as integral parts of ecosystems, in so doing placing disease emergence in its proper ecological context [19, 20].

Although well intended, eliminating natural infectious agents from wildlife during management actions such as translocations may not be beneficial to the species in the long term, with recent evidence suggesting that eliminating obligate parasites for host preservation may have unintended consequences for the health and long-term natural resistance of host species in the wild [21, 22]. However, although rare, diseases have occasionally emerged with links to a translocation event. A reintroduction of the green and golden bell frog *Litoria aurea* in New South Wales, Australia failed due to the emergence of amphibian chytrid fungus *bartrachochyhiurn dendrobatidis* [23]. Two recently translocated populations of tieke/South Island saddleback (*Philesturnus carunculatus*) in New Zealand declined by up to 60% as a result of concurrent infections with avian malaria, coccidiosis and avian pox virus, although the population did quickly recover [24–26]. The short- and long-term risk of disease emergence needs to be balanced against the clear benefits deriving from translocations. Like most threatened populations, many of New Zealand’s endemic bird species could be vulnerable to infectious disease due to their low population size, limited genetic diversity and isolated evolution [27, 28]. When diseases do emerge in naïve populations, it can result in high levels of mortality leading to significant population declines and increasing extinction risk [29]. Understanding the infectome of a species across source and release habitats may be an important step in ensuring translocations are successful not just for the host, but at a broader ecosystem level.

Toutouwai (North Island robins, *Petroica longipes*) are small, endemic passerines of New Zealand. They belong to the ‘Australasian robin’ family *Petroicidae* that also include the kakaruwai (South Island Robin, *P. australis*) and the karure/kakaruia (Chatham Islands black robin, *P. traversi*). There is very limited knowledge of the infectome of this family, with only one metagenomic study including the karure [30], and two studies including the kakaruwai [31, 32]. To our knowledge, the infectome of the toutouwai has never been studied. The 41 toutouwai studied here were translocated 100 km within the North Island of New Zealand, from Bushy Park Tarapuruhi to establish a new population in Turitea Reserve near Palmerston North [33]. Bushy Park Tarapuruhi is a small (100 ha), fenced sanctuary near Whanganui containing a range of rare and threatened native species, most of which were introduced there via translocations. Turitea Reserve is a large (4000 ha) unfenced reserve containing a diverse bird assemblage [33], including some species absent from Bushy Park (e.g. Whitehead/pōpokotea *Mohoua albicilla* and Rifleman/Tītitipounamu *Acanthisitta chloris*).

Herein, we used total RNA sequencing (i.e., metatranscriptomics) to characterise the cloacal infectome of toutouwai, the first such analysis of this species or of any translocated population in New Zealand. Using these data, we describe the infectome of pooled samples from 41 individuals and discuss the implications of the microbes detected for the newly translocated population.

## Methods

### Animal sampling

Fieldwork was undertaken at Bushy Park Tarapuruhi, Whanganui, New Zealand (− 39.797, 174.928) on the 19th and 20th April 2021. For the purposes of translocation, 41 toutouwai (17 males, 24 females) were caught at the approximately 100-ha site using traps baited with meal worms. Once caught, the birds were weighed, measured (tarsus and wing length) and banded. A cloacal swab was taken using a sterile nylon mini-tip flocked swab FLOQswab™ (Copan), then cut using scissors sterilised with 70% alcohol, and placed into a tube with 1 ml of RNAlater. Samples were kept cold (< 4 °C) for the duration of the fieldwork, then frozen at − 80 °C.

### Metatranscriptomic sequencing

RNA was extracted using the RNeasy plus mini extraction kit (Qiagen) and QIAshredders (Qiagen). The tube containing the swab in RNAlater was thawed and placed in 600ul of extraction buffer using sterile forceps. The swab and buffer were vortexed at maximum speed for 2 minutes before being placed into a QIAshredder and centrifuged at maximum speed for 5 minutes. Avoiding the cell debris pellet the flowthrough was retained and used in the extraction following the standard protocol in the kit. The RNA was eluted into 50ul of sterile water.

To increase the concentration of RNA, extractions were randomly pooled into groups of 3–5 animals for sequencing, using 25ul of each extraction in each pool. This pooled RNA was concentrated using the NucleoSpin RNA Clean-up XS, Micro kit for RNA clean up and concentration (Machery-Nagel). The concentrated RNA was eluted into 20ul of sterile water. The Stranded Total RNA Prep with Ribo-Zero Plus (Illumina) kit was used to prepare the cDNA libraries for paired-end sequencing on the Illumina Novaseq 6000 platform. Using two lanes, each sample was sequenced once on each lane and the reads were combined from both lanes for each sample.

### Quality control and assembly

Trimmomatic (0.38) was used to trim the Nextera paired-end adapters [34]. Using a sliding window approach, bases below a quality of 5 were trimmed with a window size of 4. Bases were cut if below a quality of 3 at the beginning and end of the reads. Bbduk in BBtools (bbmap 37.98) was used to remove sequences below an average quality of 10 or less than 100 nucleotides in length [35]. Trinity (2.8.6) was used for de novo read assembly [36].

### Virus identification

Viruses were identified using Blastn (blast+ 2.9.0) and Diamond Blastx (diamond 2.0.9) by comparing the assembled contigs to the NCBI nucleotide database (nt) and non-redundant protein database (nr) [37, 38]. Contigs with hits to viruses and with an open reading frame greater than 300 nucleotides (for nr hits) were retained. As such, we detected viral contigs ranging in size from 300 to 12,034 nucleotides. To avoid false-positives, sequence similarity cut-off values of 1E-5 and 1E-10 were used for the nt and nr databases, respectively. Bowtie2 (2.2.5) was used to estimate viral abundance [39], expressed as the number of reads per million (read count divided by the total number of reads in the library, multiplied by 1 million) to account for differences in read depth between libraries. Viruses were assumed to be contamination due to index-hopping from another library if the total read count was < 0.1% of the read count in the other library, and they were > 99% identical at the nucleic acid level. No viruses were identified that met this criteria. A blank negative control library (a sterile water and reagent mix) was sequenced alongside the samples. Any viruses found in this library were assumed to be reagent contamination and removed from all sample libraries.

Phylogenetic trees were estimated for viruses belonging to viral families that infect vertebrates, using the non-structural polyprotein or RNA dependent RNA polymerase (RdRp) gene. The L-INS-i algorithm in MAFFT (7.402) was used to align amino acid sequences [40], and Trimal (1.4.1) to trim the alignments [41], using a gap threshold of 0.9 and at least 20% of the sequence conserved (Additional Table 1). IQ-TREE (1.6.12) was used to infer individual maximum likelihood phylogenetic trees for each virus family [42], with the best-fit substitution model determined by the program and employing the approximate likelihood ratio test with 1000 replicates to assess node robustness. Phylogenetic trees and figures were produced using APE (5.4) and ggtree (2.4.1) in R [43, 44].

### Bacteria, fungal and parasite screening

Eukaryotic, bacterial and fungal diversity was characterized using CCMetagen (v 1.2.4) and the NCBI nucleotide database (nt) [38, 45]. The results were manually screened to identify known pathogens, and sequences of interest were blasted to the NCBI nucleotide database (nt) to identify the gene and screen for false positives. Contigs were aligned to reference genes (closest blast match) using Geneious [46] and the consensus sequence was used in phylogenetic analysis. 16S and 18S ribosomal RNA (rRNA) nucleotide sequences were aligned in MAFFT using the G-INS-i algorithm with no trimming, and maximum likelihood trees were created using IQ-TREE as described above.

## Results

We took cloacal swabs of 41 individual toutouwai sampled from a single geographic location – Bushy Park Tarapuruhi, Whanganui. The 41 toutouwai samples were pooled into nine libraries for total RNA sequencing, from which we generated an average of 54 million reads and a total of 489 million reads (Additional Table 2). These RNA reads were analysed using a metatranscriptomic protocol to characterize the RNA microbiome and virome.

**Fig. 1:**
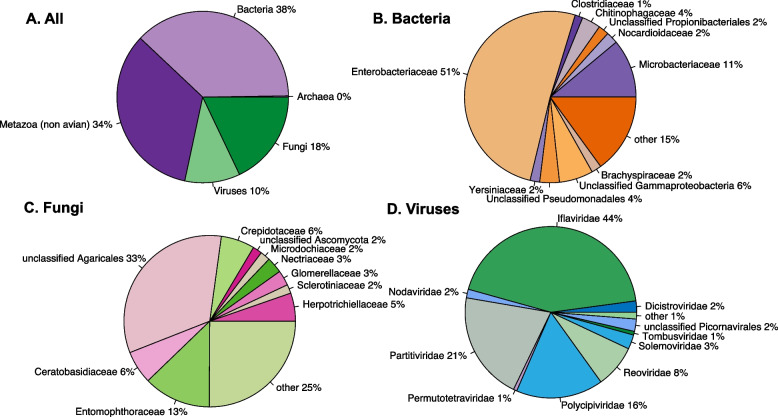
Pie charts detailing the toutouwai cloacal infectome. The size of each slice represents the relative abundance of the group in question. **A** The proportion of RNA contigs with hits to non-avian metazoan species, bacteria, viruses and fungi, following removal of avian hits. **B-D** The proportion of RNA with hits to the ten most abundant bacteria, fungi and virus families. The remaining families are grouped into ‘other’. The percentage of the total abundance (the sum of all pools) is shown, rounded to the nearest whole number

All libraries consisted almost entirely of bird RNA (accounting for 97–99.9% of assembled contigs), with most of the remaining sequence reads associated with commensal bacteria, host diet or the environment (such as soil associated fungi). When bird RNA was excluded, bacterial RNA accounted for over one third of the total (Fig. 1a), consisting predominately of Enterobacteriaceae, including non-pathogenic strains of *Escherichia* and *Shigella* (Fig. 1b). RNA from non-avian metazoa also made up a high proportion, which included arthropods, annelids, molluscs, nematodes and platyhelminths, reflecting the insectivorous diet of the host (Fig. 1a). Fungal reads consisted of predominately *Entomophthoraceae* and *Agaricales*, likely with soil origins (Fig. 1c). The most abundant viral families were those associated with fungi or invertebrates, namely *Partitiviridae*, *Iflaviridae* and *Polycipiviridae* (Fig. 1d)*.* We did not identify any known avian diseases, including avian malaria and pox virus.

### Viruses

In total, we identified viruses from 28 different viral families (Additional Table 3). Of these, 14 families (111 viruses) were primarily associated with invertebrates, nine (64 viruses) have plants and fungi as their usual hosts, and two families (five viruses) are associated with bacteria. The remaining three families – the *Caliciviridae, Hepeviridae,* and *Picornaviridae –* include viruses that infect vertebrates and hence may be directly associated with the avian hosts studied here, although no known avian viral diseases were identified. Detailed phylogenetic analysis was then performed on each of these families of vertebrate viruses which we now describe in turn.

#### Caliciviridae

**Fig. 2:**
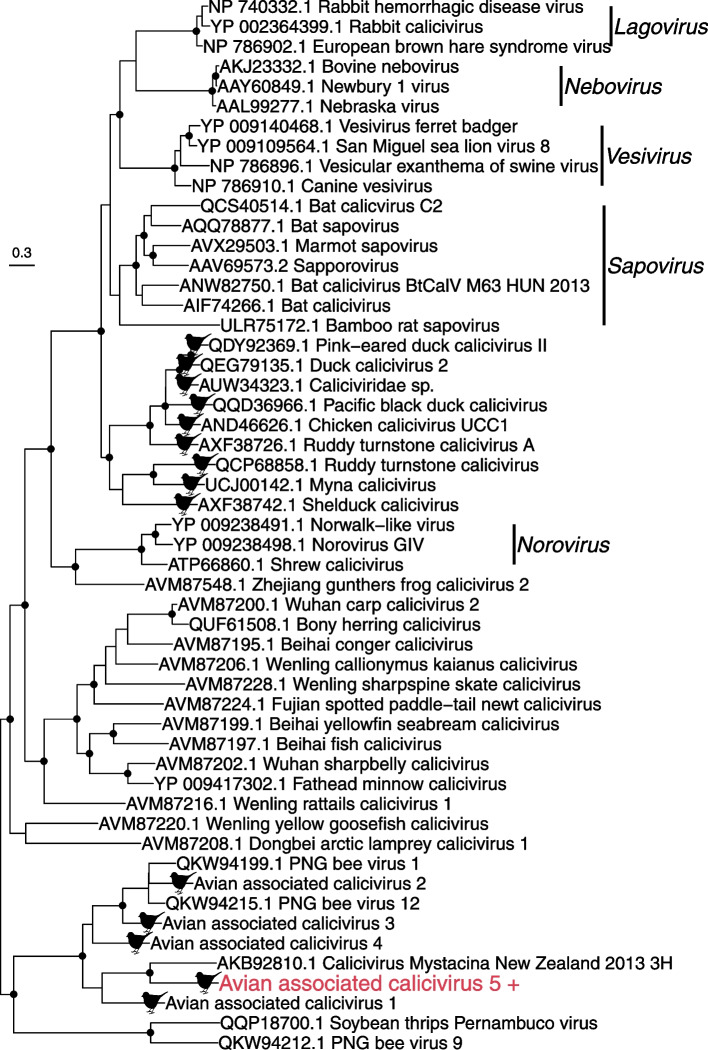
Phylogeny of the *Caliciviridae* (representative viruses only) based on the non-structural polyprotein (alignment length of 1132 amino acids post trimming). The virus obtained this study is shown in red and has a ‘+’ after the name. Viruses found in birds are marked with a bird silhouette. Related viruses are shown in black. Black circles on nodes show bootstrap support values of more than 90%. Branches are scaled according to the number of amino acid substitutions per site, shown in the scale bar. The tree is midpoint rooted for purposes of clarity only

The *Caliciviridae* are a family of positive-sense, single-stranded RNA viruses, commonly associated with vertebrates. We identified one novel calici-like virus, with an abundance of 155 reads per million (RPM), that fell into a clade distinct from all currently designated genera (Fig. 2). This virus – provisionally denoted avian associated calici-like virus 5 - was most closely related to the partial helicase of calicivirus Mystacina/New Zealand/2013/3H found in the New Zealand lesser short-tailed bat/pekapeka (*Mystacina tuberculata)* [47]. These two viruses only exhibit 43.3% amino acid identity to the non-structural polyprotein, suggesting a distant evolutionary separation, albeit one that likely occurred in New Zealand. They also cluster with two viruses found in bees (PNG bee virus 1 and 12) in Papua New Guinea [48], and four other avian viruses found in blackbirds (*Turdus merula)* and dunnocks (*Prunella modularis)* in New Zealand [32]. Given the mixture of invertebrate and vertebrate hosts in this clade it is difficult to conclusively determine the true host species.

#### Hepeviridae

**Fig. 3:**
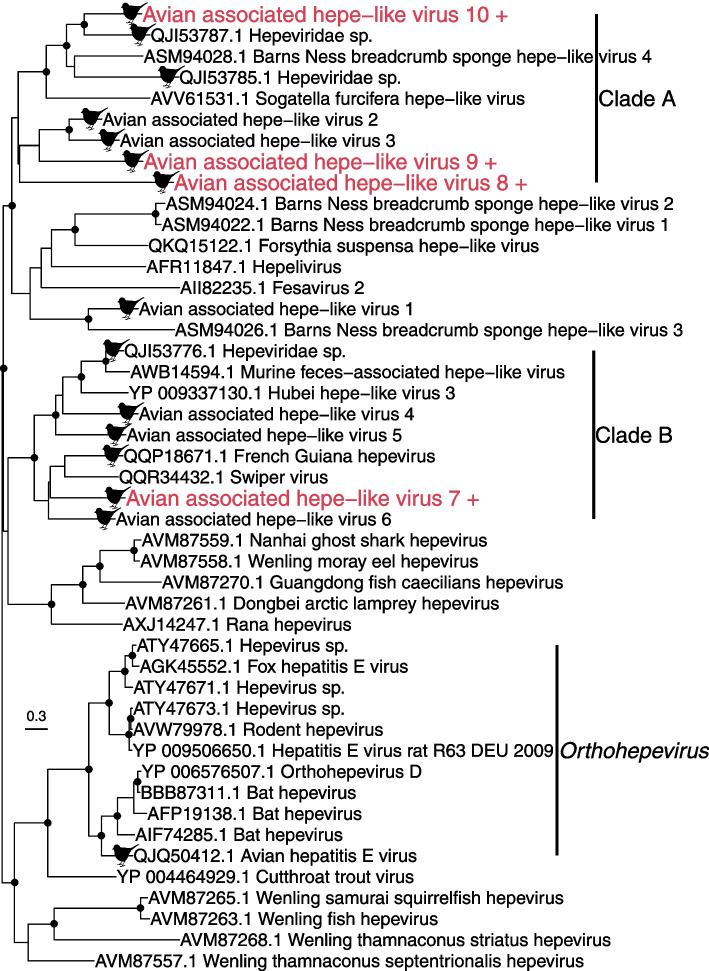
Phylogeny of the *Hepeviridae* (representative viruses only) based on the non-structural polyprotein (alignment length of 906 amino acids post trimming). The viruses from this study are shown in red and have a ‘+’ after their name. Viruses found in birds are marked with a bird silhouette. Related viruses are shown in black. Black circles on nodes show bootstrap support values of more than 90%. Branches are scaled according to the number of amino acid substitutions per site, shown in the scale bar. The tree is midpoint rooted for purposes of clarity only

The *Hepeviridae* are a family of positive-sense, single-stranded RNA viruses associated with a variety of vertebrate and invertebrate taxa. We identified four novel hepe-like viruses that fell into clades distinct from the genus *Orthohepevirus* commonly found in vertebrates (Fig. 3). Three of these viruses – avian associated hepe-like virus 8–10 – fall into a diverse clade although with weak support (Fig. 3, clade A) with a combined abundance of 116 RPM. This clade includes viruses found in both vertebrates and invertebrates, although the viruses we identified are most closely related to Hepeviridae sp. found in birds in China, and avian associated hepe-like virus 2 and 3 – found in the bellbird (*Anthornis melanura)* and dunnock in New Zealand [32]. Avian associated hepe-like virus 7 falls into a clade with other viruses found in vertebrates with strong support (Fig. 3, clade B), including five viruses previously identified in birds. This virus also had a higher abundance (274 RPM). Three of these viruses – avian associated hepe-like virus 4, 5 and 6 – were found in New Zealand birds: in the thrush (*Turdus philomelos)* and silvereye (*Zosterops lateralis*) [32], while two others were found in birds in French Guiana [49] and China. Combined, these data suggest that the hepeviruses identified here are likely to be avian viruses. However, both clades A and B on the *Hepeviridae* phylogeny also include viruses found in invertebrates (for example, Hubei hepe-like virus 3 was found in a centipede), raising the possibility that these viruses are in fact associated with invertebrates and detected in the birds through their diet.

#### Picornaviridae

**Fig. 4:**
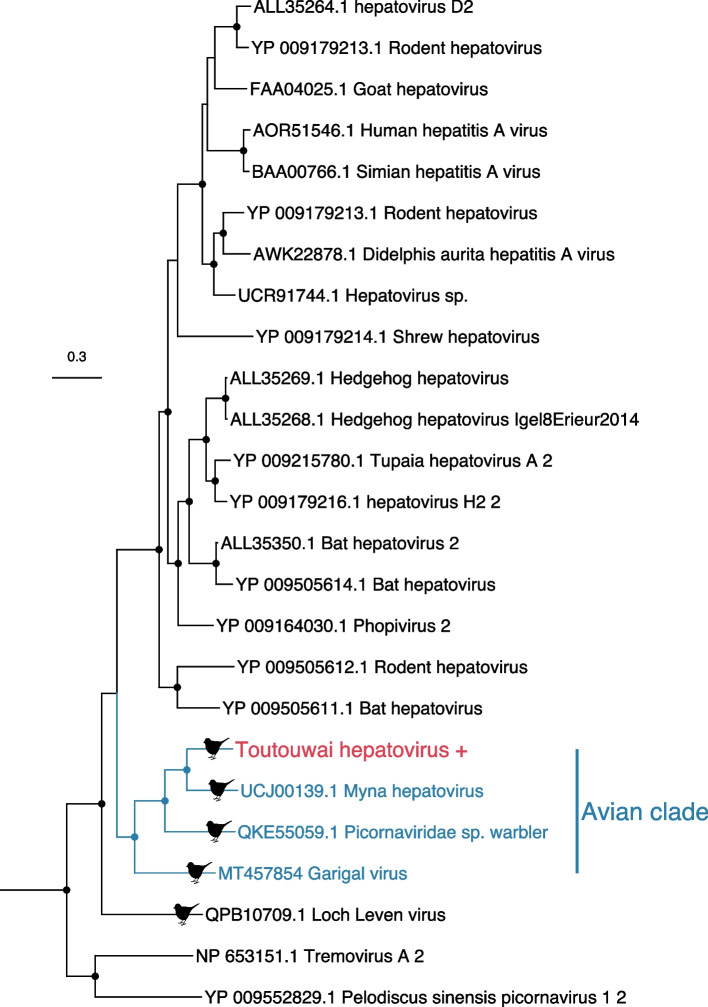
Phylogeny of the genus *Hepatovirus* (representative viruses only) based on the RNA-dependent RNA polymerase gene (alignment length of 2201 amino acids post trimming). The virus from this study (toutouwai hepatovirus) is shown in red and has a ‘+’ after the name. The avian clade is shown by the blue tips and labels. Viruses found in birds are marked with a bird silhouette. Related viruses are shown in black. Black circles on nodes show bootstrap support values of more than 90%. Branches are scaled according to the number of amino acid substitutions per site, shown in the scale bar. The tree is midpoint rooted for purposes of clarity only

The *Picornaviridae* is a family of positive-sense, single-stranded RNA viruses that infect both vertebrates and invertebrates. We identified 14 novel picorna-like viruses that fell in a variety of phylogenetic locations within this diverse family. Although most of these viruses were likely associated with host diet as they were more closely related to viruses infecting invertebrates, plants, fungi, (Additional Fig. 1), we identified one virus – toutouwai hepatovirus – in two libraries that fell within the genus *Hepatovirus* (Fig. 4), although with an abundance of only 0.4 RPM. This virus clusters in a clade with three other bird viruses found in a yellow-browed warbler (*Abrornis inornata*), common myna (*Acridotheres tristis*) and rainbow lorikeet (*Trichoglossus moluccanus*) [50, 51], suggesting it is a bona fide avian virus.

### Brachyspira

**Fig. 5:**
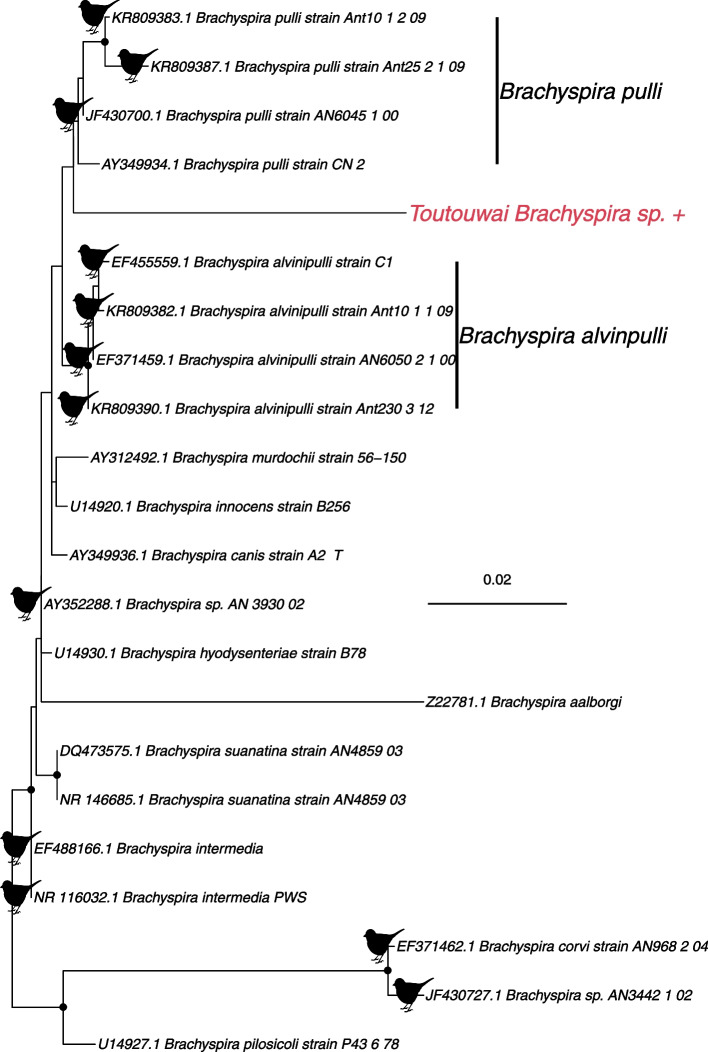
Phylogeny of the bacterial genus *Brachyspira*, based on the 16S rRNA gene (alignment length of 1542 nucleotides). The bacterium from this study (*toutouwai Brachyspira* sp.) is shown in red and has a ‘+’ after the name. Spirochetes found in birds are marked with a bird silhouette. Black circles on nodes show bootstrap support values of more than 90%. Branches are scaled according to the number of nucleotide substitutions per site, shown in the scale bar. The tree is midpoint rooted for purposes of clarity only

As well as viruses, we identified a number of bacterial taxa. Although most of the species identified were likely commensals, our data contained Brachyspira - a spirochete that infects a variety of animals including birds and mammals. Specifically, we identified 13 contigs belonging to the bacterial family *Brachyspiraceae*, of which six were 16S rRNA, and six were 23S rRNA, with an abundance of 54 RPM. The remaining contig had closest hits to hypothetical genes such that it’s true status is uncertain. The 16S rRNA contigs were aligned to a reference sequence to create a 1068 bp partial gene sequence. Phylogenetic analysis (Fig. 5) indicated that the toutouwai Brachyspira was most closely related (96% nucleotide identity) to *Brachyspira pulli* and *Brachyspira alvinpulli*, but with a relatively long branch length. Both these species have been previously detected in birds, with *B. alvinipulli* a known pathogen and *B. pulli* considered commensal in chickens [52].

### Coccidian parasite

**Fig. 6:**
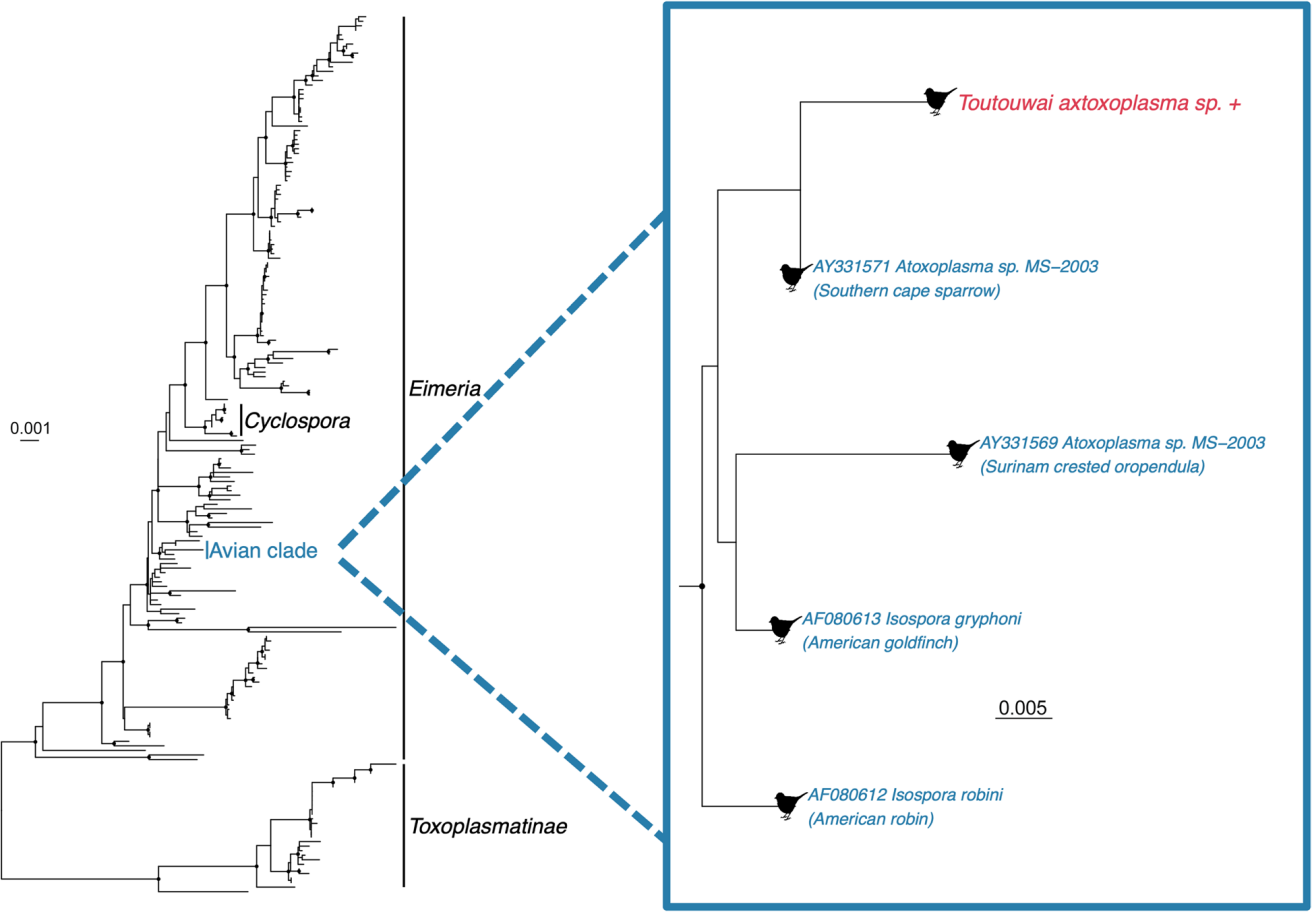
Phylogeny of the coccidian parasite family *Eimeriidae* based on the 18S rRNA gene (alignment length of 3084 nucleotides). The coccidium from this study – denoted toutouwai axtoxoplasma sp. - is shown in red and has a ‘+’ after the name. The avian clade (representative sequences only) is shown in the inset and are marked with a bird silhouette. Black circles on nodes show bootstrap support values of more than 90%. Branches are scaled according to the number of nucleotide substitutions per site, shown in the scale bar. The tree is midpoint rooted for purposes of clarity only

Also of note, we identified 35 contigs belonging to the eukaryotic order Eurococcidia, with coccidia representing common protozoan intestinal parasites of birds [53]. These contigs were primarily 28S rRNA (25 contigs) and 18S rRNA (6 contigs), with an abundance of 324 RPM. The 18S rRNA contigs were aligned to a reference sequence to create a 762 bp partial gene sequence. Phylogenetic analysis of these data reveals that the coccidia identified here is part of a well-supported clade of four avian coccidia from avian Isospora and Atoxoplasma suggesting that it is a bona fide avian parasite (Fig. 6, Additional Fig. 2). This avian clade includes a further 18 taxa from birds that were not included in our tree due to the short sequence length (< 250 nucleotides). As observed previously [54] the Isospora, Atoxoplasma and Eimeria are monophyletic, and this avian clade appears to sit broadly within a larger Eimeria grouping. This was the only protozoan of note observed here.

## Discussion

We characterised the cloacal infectome of 41 translocated toutouwai. Importantly, we found no evidence for the presence of any known avian diseases, including avian malaria and pox virus, which is unsurprising as all the birds sampled appeared healthy. Although our sample size is necessarily small in comparison to the total robin source population, the absence of any known disease-causing microorganisms indicates the risk is low of transferring disease to the destination population, or to other species at the release site. However, we did detect a number of presumably non-pathogenic microorganisms, further demonstrating that a large majority of microbial species likely do not cause disease in their hosts [19, 20]. In particular, we identified a number of putative avian viruses of interest including a novel hepatovirus, calici-like virus and four novel hepe-like viruses, as well as a new spirochete and coccidian parasite.

Of particular note was the identification of a novel hepatovirus species that fell within a clade with two viruses sampled from birds in Australia, as well as with a bird from China. Although hepatovirus A causes disease in humans, other hepatovirus species have been found in seemingly healthy birds and mammals, suggesting it may be routinely non-pathogenic in wildlife [50, 55]. Hepatoviruses were originally thought only to occur in primates [56], although recent studies have detected hepatoviruses in many mammalian species including bats, rodents, seals and marsupials, indicating that their host range is far wider than originally realised [55, 57, 58]. Based on these findings, hepatoviruses are now thought to have evolved in small mammals before jumping to humans and primates [55]. However, recent studies have also identified hepatoviruses in birds [50, 59], and our phylogenetic analysis suggests that the avian viruses sit basal to those viruses found in small mammals, both with midpoint rooting and using the closely related genus *Tremovirus* as an outgroup. This phylogenetic pattern suggests that birds, rather than mammals, may be the original host, and is consistent with long-term virus-host co-divergence during vertebrate evolution, although cross-species transmission is commonplace within mammal hepatoviruses [55, 57].

We identified a divergent calici-like virus and four divergent hepe-like viruses. All closely related viruses were found in seemingly healthy animals, suggesting that these viruses do not commonly cause disease. The calici-like virus was most closely related to viruses found in the New Zealand short-tailed bat/pekapeka [47] and to four viruses found in New Zealand birds. This may indicate the presence of a divergent New Zealand-specific clade, with a cross-species transmission event from birds to bats, or vice versa, although this will need to be confirmed with more extensive sampling. Interestingly, these caliciviruses have now been observed in both endemic species (toutouwai and pekapeka) as well as the blackbird and dunnock that were introduced to New Zealand from England in the 1800s, suggesting recent cross-species transmission to these species within New Zealand, although the direction of transmission is unknown. However, these viruses also cluster with two viruses found in bees in Papua New Guinea [48]. This raises the possibility that the viruses found in the bat and birds are in fact of dietary origin, particularly as these animals are all either entirely or partially insectivorous. Sampling of New Zealand invertebrates may shed further light on the origin of these viruses. Toutouwai are predominately insectivorous, but also occasionally eat fungi and fruit. Indeed, many of the viruses identified in this study appear to reflect this diverse diet, with invertebrate infecting viruses exhibiting the highest diversity at the family level (*n* = 14), followed closely by plant and fungal viruses (*n* = 9).

One of the hepe-like viruses we identified fell into a clade of vertebrate viruses, containing those found in the house mouse (*Mus musculus),* red fox (*Vulpes vulpes*) [60] and five different bird species from China, French Guiana [49] and New Zealand [32]. However, this clade also includes a virus found in the house centipede (*Scutigera coleoptrata*), again raising some doubt about the host provenance of these viruses. More broadly, this demonstrates the difficulty in determining the true host of viruses from metagenomic data, particularly when they cluster with viruses found in a wide range of host taxa.

We detected a spirochete bacterium that may be most closely related to *Brachyspirales pulli* and *B. alvipulli.* Wild birds, particularly waterfowl, are considered reservoirs for many *Brachyspirales* spp. [61], and these bacteria have a wide geographic range [62]. Although spirochete infections have caused disease in wild geese [63], *Brachyspirales* spp. are regularly found in healthy individuals [64, 65] such that their status as pathogens is unclear. Further research is required to determine whether this is a disease risk in toutouwai, although this appears unlikely.

Finally, we detected a coccidian parasite from the family *Eimeriidae* belonging to a clade found in birds [66]. Coccidia are commonly detected in many different bird species worldwide (including the toutouwai), with evidence of cross-species transmission between passerine species [25, 66]. At low levels, coccidia are thought to have little impact on the host, although severe infections have occasionally caused mortalities in multiple endemic birds in New Zealand, particularly with additional stressors such as captivity and translocations [25]. However, eradicating naturally present parasites may disadvantage translocated birds if they are re-exposed when released [67]. Proactive measures such as reducing stress, strict hygiene, low stocking densities and a low dose of prophylactic treatment when in captivity can reduce the excretion of oocysts, which have been shown to prevent severe disease and improve translocation success while preventing a complete eradication of the parasite [67, 68]. Therefore, the presence of coccidia should be considered as a potential disease risk, particularly if translocations involve a period of captivity with high stocking densities. For this translocation of toutouwai the period of captivity was less than 48 hours in individual boxes, which will have reduced the risk of severe infection developing.

## Conclusions

Before conducting a translocation it is beneficial to conduct disease surveillance of the source population as well as possible pathogen reservoirs in the new location [18], but also to build an understanding of the natural infectome across multiple sites over time. Not only will this help to assess the risk of disease emergence following translocation, but it also enables the discovery of novel viral and microbial species, including those in native bird species that are currently poorly understood. There is often valid concern about the risk translocations pose for the spread of infectious disease. However, it is also important to recognise that translocations may act as an important tool for the restoration of complete ecosystems and may produce benefits in the long-term. Translocations of potentially unhealthy individuals from modified or poor-quality habitats may spread disease, so that knowing the viral composition at a source site prior to a translocation is of great importance. In addition, gathering baseline data on healthy individuals may allow future disease emergence to be put in its proper context [19, 69]. In other cases, translocations of individuals from remnant, intact ecosystems may provide a means of restoring important microbial and viral communities, which can in turn promote long-term resilience to the translocated population and other co-occurring species, and lead to an improvement in overall ecosystem health.

## Supplementary Information

**Additional file 1: Fig. 1.** Phylogeny of the *Picornaviridae* (representative viruses only) based on the RdRp (alignment length of 2201 amino acids). The viruses from this study are shown in blue and have a ‘+’ after the name. Related viruses are shown in black. Black circles on nodes show bootstrap support values of more than 90%. Branches are scaled according to the number of amino acid substitutions per site, shown in the scale bar. The tree is midpoint rooted for purposes of clarity only.

**Additional file 2: Fig. 2.** Phylogeny of the coccidian parasite family *Eimeriidae* based on the 18S rRNA gene (alignment length of 3084 nucleotides). The coccidium from this study – denoted toutouwai axtoxoplasma sp. - is shown in blue and has a ‘+’ after the name. Black circles on nodes show bootstrap support values of more than 90%. Branches are scaled according to the number of nucleotide substitutions per site, shown in the scale bar. The tree is midpoint rooted for purposes of clarity only.

**Additional file 3: Table 1.** Details of the sequence alignments used to estimate the phylogenetic trees for each genus/family. The % pairwise identity refers to the percentage of pairwise residues that are identical in the alignment, excluding gap-gap residues.

**Additional file 4: Table 2.** Information about each library pool: the number of individual samples and total number of reads.

**Additional file 5: Table 3.** List of the viral families identified, their genome type, the abundance expressed as reads per million (RPM, read count divided by the total number of reads in the library, multiplied by one million) and the number of viruses found in each library.

## Data Availability

The sequencing data supporting the conclusions of this article have been deposited in the Sequence Read Archive (SRA) under the accession numbers SAMN29543946–54. Consensus sequences have been submitted to NCBI/GenBank and assigned accession numbers ON968934–56.

## Abbreviations

NCBI: National Center for Biotechnology Information
rRNA: Ribosomal ribonucleic acid
RPM: Reads per million

## References

[1] IUCN (2013). Guidelines for reintroductions and other conservation translocations. Version 10 IUCN Species Survival Commission Gland, Switzerland.

[2] Seddon PJ (2010). From reintroduction to assisted colonization: moving along the conservation translocation spectrum. Restor Ecol.

[3] Batson WG, Gordon IJ, Fletcher DB, Manning AD (2015). Translocation tactics: a framework to support the IUCN guidelines for wildlife translocations and improve the quality of applied methods. J Appl Ecol.

[4] Lloyd B, Powlesland R (1994). The decline of kakapo *Strigops habroptilus* and attempts at conservation by translocation. Biol Conserv.

[5] Morris SD, Brook BW, Moseby KE, Johnson CN (2021). Factors affecting success of conservation translocations of terrestrial vertebrates: a global systematic review. Global Ecol Conserv.

[6] El Alqamy H, Senn H, Roberts M-F, McEwing R, Ogden R (2012). Genetic assessment of the Arabian oryx founder population in the emirate of Abu Dhabi, UAE: an example of evaluating unmanaged captive stocks for reintroduction. Conserv Genet.

[7] Walters JR, Derrickson SR, Fry DM, Haig SM, Marzluff JM, Wunderle JM (2008). Status of the California condor and efforts to achieve its recovery.

[8] Ewen JG, Acevedo-Whitehouse K, Alley MR, Carraro C, Sainsbury AW, Swinnerton K (2012). Empirical consideration of parasites and health in reintroduction. Reintroduction Biology: Integrating Science and Management.

[9] Sainsbury AW, Vaughan-Higgins RJ (2012). Analyzing disease risks associated with translocations. Conserv Biol.

[10] Armstrong DP, Seddon PJ (2008). Directions in reintroduction biology. Trends Ecol Evol.

[11] Rózsa L, Vas Z (2015). Co-extinct and critically co-endangered species of parasitic lice, and conservation-induced extinction: should lice be reintroduced to their hosts?. Oryx..

[12] Hassell JM, Begon M, Ward MJ, Fèvre EM (2017). Urbanization and disease emergence: dynamics at the wildlife–livestock–human interface. Trends Ecol Evol.

[13] Berger-Tal O, Blumstein D, Swaisgood RR (2020). Conservation translocations: a review of common difficulties and promising directions. Anim Conserv.

[14] Alley M, Gartrell B (2019). Wildlife diseases in New Zealand: recent findings and future challenges. N Z Vet J.

[15] Sherman J, Unwin S, Travis DA, Oram F, Wich SA, Jaya RL (2021). Disease risk and conservation implications of orangutan translocations. Front Vet Sci.

[16] Letty J, Marchandeau S, Reitz F, Clobert J, Sarrazin F (2002). Survival and movements of translocated wild rabbits (*Oryctolagus cuniculus*). Game Wildlife Sci.

[17] Hing S, Narayan EJ, Thompson RA, Godfrey SS (2016). The relationship between physiological stress and wildlife disease: consequences for health and conservation. Wildlife Res.

[18] Parker KA, Brunton DH, Jakob-Hoff R (2006). Avian translocations and disease; implications for New Zealand conservation. Pac Conserv Biol.

[19] French RK, Holmes EC (2020). An ecosystems perspective on virus evolution and emergence. Trends Microbiol.

[20] Hudson PJ, Dobson AP, Lafferty KD (2006). Is a healthy ecosystem one that is rich in parasites?. Trends Ecol Evol.

[21] Dougherty ER, Carlson CJ, Bueno VM, Burgio KR, Cizauskas CA, Clements CF (2016). Paradigms for parasite conservation. Conserv Biol.

[22] Spencer HG, Zuk M (2016). For host's sake: the pluses of parasite preservation. Trends Ecol Evol.

[23] Stockwell M, Clulow S, Clulow J, Mahony M (2008). The impact of the amphibian chytrid fungus *Batrachochytrium dendrobatidis* on a green and golden bell frog *Litoria aurea* reintroduction program at the hunter wetlands Centre Australia in the hunter region of NSW. Aust Zool.

[24] Alley M, Hale K, Cash W, Ha H, Howe L (2010). Concurrent avian malaria and avipox virus infection in translocated South Island saddlebacks (*Philesturnus carunculatus carunculatus*). N Z Vet J.

[25] Schoener E, Alley M, Howe L, Castro I (2013). Coccidia species in endemic and native New Zealand passerines. Parasitol Res.

[26] Hale KA, Briskie JV (2009). Rapid recovery of an island population of the threatened South Island saddleback *Philesturnus c. carunculatus* after a pathogen outbreak. Bird Conserv Int.

[27] Robertson HA, Baird KA, Elliott GP, Hitchmough RA, McArthur NJ, Makan TD (2021). Conservation status of birds in Aotearoa New Zealand, 2021. New Zealand Threat Classification Series 36.

[28] Wikelski M, Foufopoulos J, Vargas H, Snell H (2004). Galápagos birds and diseases: invasive pathogens as threats for island species. Ecol Soc.

[29] Russell RE, DiRenzo GV, Szymanski JA, Alger KE, Grant EH (2020). Principles and mechanisms of wildlife population persistence in the face of disease. Front Ecol Evol.

[30] Sikorski A, Massaro M, Kraberger S, Young LM, Smalley D, Martin DP, Varsani A (2013). Novel myco-like DNA viruses discovered in the faecal matter of various animals. Virus Res.

[31] Custer JM, Robyn W, Taylor H, Schmidlin K, Fontenele RS, Stainton D, Kraberger S, Briskie JV, Varsani A (2022). Diverse single-stranded DNA viruses identified in New Zealand (Aotearoa) south island robin (*Petroica australis*) fecal samples. Virology..

[32] French RK, Filion A, Niebuhr CN, Holmes EC (2022). Metatranscriptomic comparison of viromes in endemic and introduced passerines in New Zealand. Viruses..

[33] Stone Z, Parker KA (2022). Unmanned aerial vehicle (UAV) activity elicits little to no response from New Zealand forest birds during wildlife monitoring. Notornis..

[34] Bolger AM, Lohse M, Usadel B (2014). Trimmomatic: a flexible trimmer for Illumina sequence data. Bioinformatics..

[35] Bushnell B (2016). BBMap short read aligner.

[36] Grabherr MG, Haas BJ, Yassour M, Levin JZ, Thompson DA, Amit I (2011). Trinity: reconstructing a full-length transcriptome without a genome from RNA-Seq data. Nat Biotechnol.

[37] Buchfink B, Xie C, Huson DH (2015). Fast and sensitive protein alignment using DIAMOND. Nat Methods.

[38] Camacho C, Coulouris G, Avagyan V, Ma N, Papadopoulos J, Bealer K (2009). BLAST+: architecture and applications. BMC Bioinformat.

[39] Langmead B, Salzberg S (2013). Fast gapped-read alignment with bowtie 2. Nat Methods.

[40] Katoh K, Standley DM (2013). MAFFT multiple sequence alignment software version 7: improvements in performance and usability. Mol Biol Evol.

[41] Capella-Gutiérrez S, Silla-Martínez JM, Gabaldón T (2009). trimAl: a tool for automated alignment trimming in large-scale phylogenetic analyses. Bioinformatics..

[42] Nguyen L-T, Schmidt HA, Von Haeseler A, Minh BQ (2015). IQ-TREE: a fast and effective stochastic algorithm for estimating maximum-likelihood phylogenies. Mol Biol Evol.

[43] Yu G, Smith DK, Zhu H, Guan Y, Lam TTY (2017). Ggtree: an R package for visualization and annotation of phylogenetic trees with their covariates and other associated data. Methods Ecol Evol.

[44] Paradis E, Schliep K (2019). ape 5.0: an environment for modern phylogenetics and evolutionary analyses in R. Bioinformatics..

[45] Marcelino VR, Clausen PT, Buchmann JP, Wille M, Iredell JR, Meyer W (2020). CCMetagen: comprehensive and accurate identification of eukaryotes and prokaryotes in metagenomic data. Genome Biol.

[46] Kearse M, Moir R, Wilson A, Stones-Havas S, Cheung M, Sturrock S (2012). Geneious basic: an integrated and extendable desktop software platform for the organization and analysis of sequence data. Bioinformatics..

[47] Wang J, Moore NE, Murray ZL, McInnes K, White DJ, Tompkins DM (2015). Discovery of novel virus sequences in an isolated and threatened bat species, the New Zealand lesser short-tailed bat (*Mystacina tuberculata*). J Gen Virol.

[48] Roberts JM, Simbiken N, Dale C, Armstrong J, Anderson DL (2020). Tolerance of honey bees to Varroa mite in the absence of deformed wing virus. Viruses..

[49] Truchado DA, Llanos-Garrido A, Oropesa-Olmedo DA, Cerrada B, Cea P, Moens MA (2020). Comparative metagenomics of Palearctic and Neotropical avian cloacal viromes reveal geographic bias in virus discovery. Microorganisms..

[50] Chang W-S, Rose K, Holmes EC (2021). Meta-transcriptomic analysis of the virome and microbiome of the invasive Indian myna (*Acridotheres tristis*) in Australia. One Health.

[51] Chang W-S, Eden J-S, Hall J, Shi M, Rose K, Holmes EC (2020). Metatranscriptomic analysis of virus diversity in urban wild birds with paretic disease. J Virol.

[52] Jansson D, Fellström C, Råsbäck T, Vågsholm I, Gunnarsson A, Ingermaa F (2008). Phenotypic and molecular characterization of *Brachyspira* spp. isolated from laying hens in different housing systems. Vet Microbiol.

[53] López G, Figuerola J, Soriguer R (2007). Time of day, age and feeding habits influence coccidian oocyst shedding in wild passerines. Int J Parasitol.

[54] Ogedengbe JD, Ogedengbe ME, Hafeez MA, Barta JR (2015). Molecular phylogenetics of eimeriid coccidia (Eimeriidae, Eimeriorina, Apicomplexa, Alveolata): a preliminary multi-gene and multi-genome approach. Parasitol Res.

[55] Drexler JF, Corman VM, Lukashev AN, van den Brand JM, Gmyl AP, Bruenink S (2015). Evolutionary origins of hepatitis a virus in small mammals. Proc Natl Acad Sci U S A.

[56] Sander A-L, Corman VM, Lukashev AN, Drexler JF (2018). Evolutionary origins of enteric hepatitis viruses. Cold Spring Harbor Perspect Med.

[57] de Oliveira CI, Sander A-L, Silva N, Moreira-Soto A, Normann A, Flehmig B (2018). A novel marsupial hepatitis a virus corroborates complex evolutionary patterns shaping the genus *Hepatovirus*. J Virol.

[58] Anthony S, St. Leger J, Liang E, Hicks A, Sanchez-Leon M, Jain K (2015). Discovery of a novel hepatovirus (phopivirus of seals) related to human hepatitis a virus. mBio..

[59] Wille M, Shi M, Hurt AC, Klaassen M, Holmes EC (2021). RNA virome abundance and diversity is associated with host age in a bird species. Virology..

[60] Campbell SJ, Ashley W, Gil-Fernandez M, Newsome TM, Di Giallonardo F, Ortiz-Baez AS (2020). Red fox viromes in urban and rural landscapes. Virus Evol.

[61] Jansson DS, Persson M, Zimmerman U, Johansson K-E (2011). Phenotypic and genetic diversity among intestinal spirochaetes (genus *Brachyspira*) in free-living wild mallards (*Anas platyrhynchos*) sampled in southern Sweden. Syst Appl Microbiol.

[62] Jansson DS, Mushtaq M, Johansson K-E, Bonnedahl J, Waldenström J, Andersson DI (2015). Intestinal spirochaetes (genus *Brachyspira*) colonise wild birds in the southern Atlantic region and Antarctica. Infect Ecol Epidemiol.

[63] Nemes C, Glávits R, Dobos-Kovács M, Ivanics É, Kaszanyitzky É, Beregszászi A (2006). Typhlocolitis associated with spirochaetes in goose flocks. Avian Pathol.

[64] Martínez-Lobo FJ, Hidalgo Á, García M, Argüello H, Naharro G, Carvajal A (2013). First identification of “*Brachyspira hampsonii*” in wild European waterfowl. PLoS One.

[65] Jansson DS, Fellström C, Johansson K-E (2008). Intestinal spirochetes isolated from wild-living jackdaws, hooded crows and rooks (genus *Corvus*): provisionally designated “*Brachyspira corvi*” sp. nov. Anaerobe..

[66] Schrenzel MD, Maalouf GA, Gaffney PM, Tokarz D, Keener LL, McClure D (2005). Molecular characterization of isosporoid coccidia (*Isospora* and *Atoxoplasma* spp.) in passerine birds. J Parasitol.

[67] McGill I, Feltrer Y, Jeffs C, Sayers G, Marshall R, Peirce M (2010). Isosporoid coccidiosis in translocated cirl buntings (*Emberiza cirlus*). Vet Rec.

[68] Ewen JG, Armstrong DP, Empson R, Jack S, Makan T, McInnes K (2012). Parasite management in translocations: lessons from a threatened New Zealand bird. Oryx..

[69] Deem SL, Karesh WB, Weisman W (2001). Putting theory into practice: wildlife health in conservation. Conserv Biol.

