# Supplementary figures and images for: Novel viral and microbial species in a translocated Toutouwai (Petroica longipes) population from Aotearoa/New Zealand
Source: One Health Outlook. 2022 Oct 12;4:16. doi: 10.1186/s42522-022-00072-z (PMC9558408; doi:10.1186/s42522-022-00072-z)

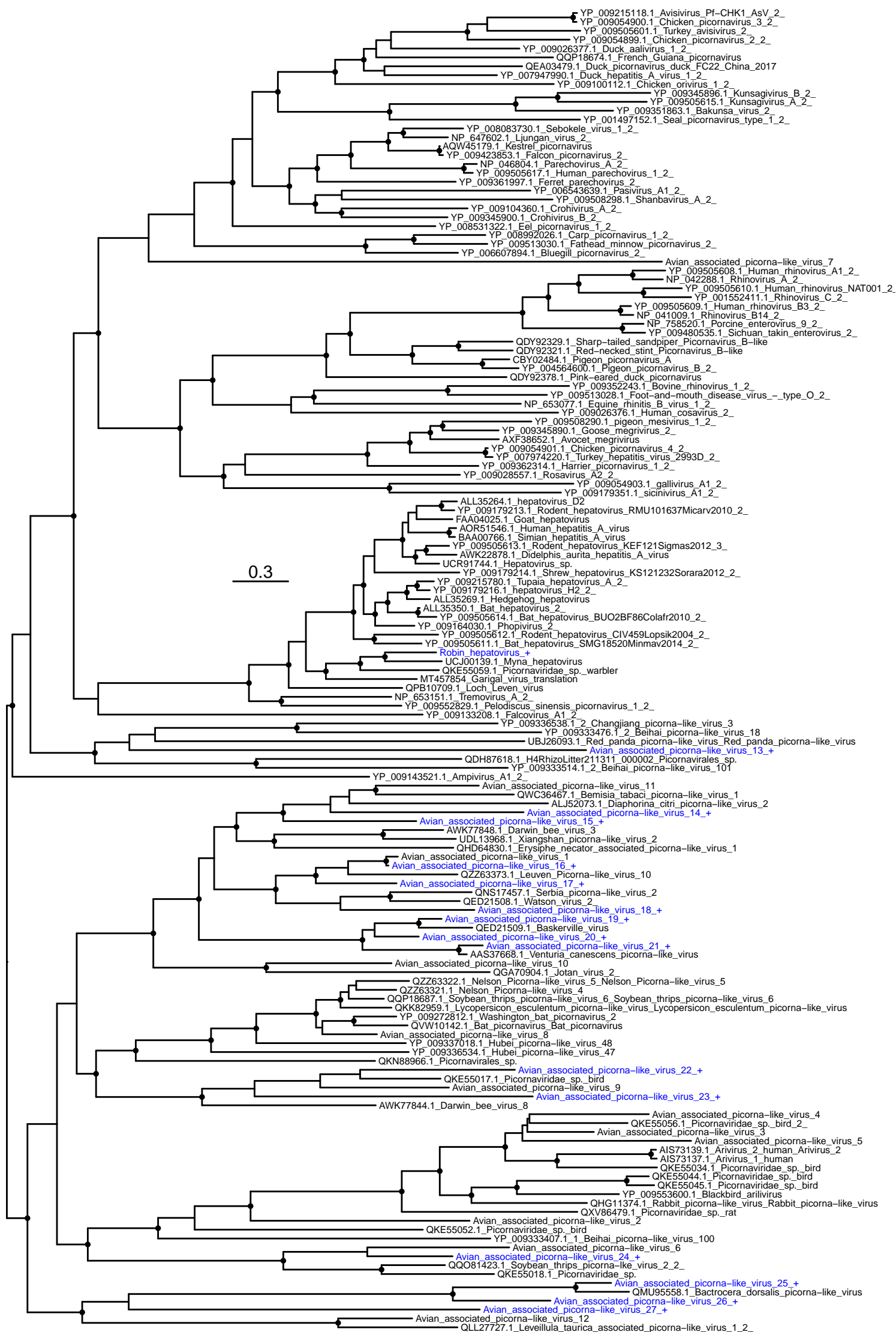

Supplement: Supplementary file 1 — Additional file 1: Fig. 1. Phylogeny of the Picornaviridae (representative viruses only) based on the RdRp (alignment length of 2201 amino acids). The viruses from this study are shown in blue and have a ‘+’ after the name. Related viruses are shown in black. Black circles on nodes show bootstrap support values of more than 90%. Branches are scaled according to the number of amino acid substitutions per site, shown in the scale bar. The tree is midpoint rooted for purposes of clarity only. [file 42522_2022_72_MOESM1_ESM.pdf]

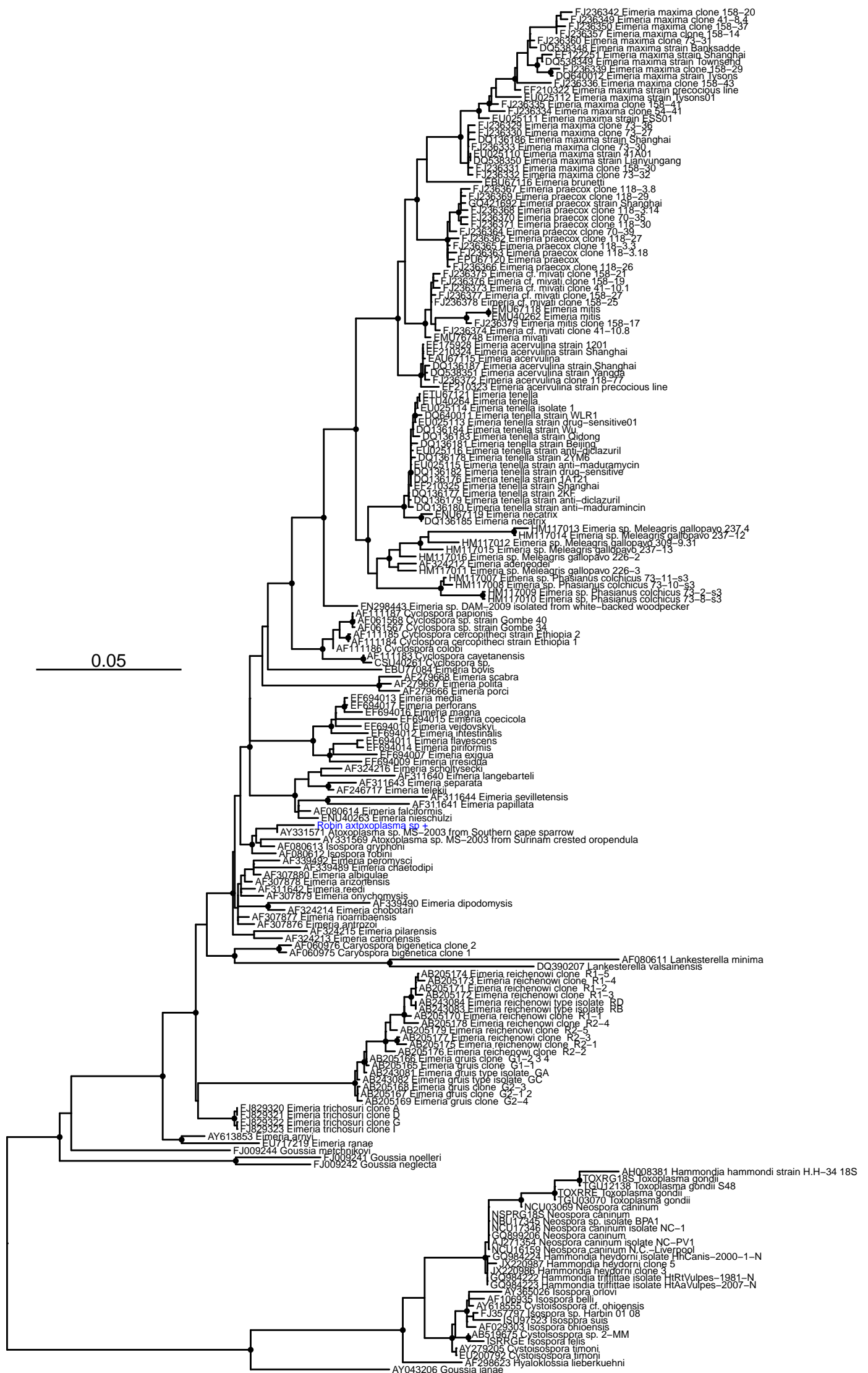

Supplement: Supplementary file 2 — Additional file 2: Fig. 2. Phylogeny of the coccidian parasite family Eimeriidae based on the 18S rRNA gene (alignment length of 3084 nucleotides). The coccidium from this study – denoted toutouwai axtoxoplasma sp. - is shown in blue and has a ‘+’ after the name. Black circles on nodes show bootstrap support values of more than 90%. Branches are scaled according to the number of nucleotide substitutions per site, shown in the scale bar. The tree is midpoint rooted for purposes of clarity only. [file 42522_2022_72_MOESM2_ESM.pdf]
